# Migraine-Specific Quality of Life Questionnaire Chinese version 2.1 (MSQv2.1-C): psychometric evaluation in patients with migraine

**DOI:** 10.1186/s12955-019-1169-y

**Published:** 2019-06-24

**Authors:** Hao-Yuan Chang, Mark P. Jensen, Chih-Chao Yang, Yeur-Hur Lai

**Affiliations:** 1 School of Nursing, College of Medicine, National Taiwan University, No.1, Sec. 1, Jen-Ai Rd, Taipei, 10051 Taiwan; 2 Department of Nursing, National Taiwan University Hospital, Taipei, Taiwan; 3 Department of Rehabilitation Medicine, University of Washington, Seattle, WA USA; 4 Department of Neurology, National Taiwan University Hospital, Taipei, Taiwan; 5 Department of Nursing, National Taiwan University Cancer Center, Taipei, Taiwan

**Keywords:** Migraine, Quality of life, Chinese version, Psychometric testing

## Abstract

**Background:**

Migraine ranks as the third most prevalent disease and the seventh most common cause of disability worldwide. To better understand the impact of migraine on the quality of life of individuals with this diagnosis, and how these might differ from one country or culture to another, reliable and valid measures of quality of life that are available in different languages are needed. To address this need, here we (1) translated the Migraine-Specific Quality of Life Questionnaire into Chinese (MSQv2.1-C), and (2) examined the psychometric properties of the measure.

**Methods:**

Forward and backward translation was conducted using four bilingual experts. One native speaker finalized the translation. Cognitive testing was performed by interviewing 11 monolingual migraineurs, and modifications were made to the MSQv2.1-C, as appropriate. Next, 174 individuals with a history of migraine completed the MSQv2.1-C, along with the SF-12, Migraine Disability Assessment Scale, and numerical rating scale s assessing pain intensity. We then evaluated the reliability and validity of the MSQv2.1-C by performing analyses to evaluate its internal consistency, test-retest reliability, convergent validity, criterion validity, and construct validity.

**Results:**

The MSQv2.1-C scales demonstrated (1) good internal consistency (Cronbach’s alpha s ≥ 0.81); (2) good 1-week test-retest reliability (intra-class coefficients ≥0.69 and Spearman’s rho correlation coefficients ≥0.74); (3) convergent validity (positive correlations with the MSQ and SF-12 scales [*rho* range = 0.27 to 0.37, *p*s < 0.05]); (4) criterion validity (negative correlations [*rho* range = − 0.51 to − 0.25, *p*s < 0.05]) between the MSQv2.1-C scales and pain-related criterion variables; and (5) construct validity (item factor loadings ranging from 0.71 to 0.96 [> 0.5]).

**Conclusions:**

The MSQv2.1-C exhibited satisfactory reliability and validity in a sample of individuals with migraine who speak Chinese. The availability of this measure will facilitate research, including cross-cultural research, on the quality of life of individuals with migraine.

## Introduction

Migraine is ranked as the third most important cause of disability in the world, affecting more than 734 million people, or about 10% of the world’s population [1, 2]. Moreover, migraine-related disability and missed workdays has significant financial costs; for example, it is associated with an annual loss of $13 billion dollars in the United States [3, 4]. Despite the high frequency and impact of migraine on quality of life, little is known regarding cross-cultural similarities and differences in the effects of migraine. In order to understand the role that culture plays on migraine and its impact, standardized measures of the effects of migraine on quality of life that have been translated into different languages are needed.

The Migraine-Specific Quality of Life Scale (MSQ) version 2.1 (MSQv2.1) [5] is one of the most widely used measures of the impact of migraine on quality of life. Although the MSQ is a commonly used measure and has been validated in English speaking populations, there is not yet a validated Chinese version of the MSQ. Given that the world’s Chinese-speaking population has reached 1.2 billion worldwide [6], the availability of a Chinese version of the MSQ would increase the utility of the measure in evaluating the effects of migraine in a large portion of the world’s population. Moreover, the availability of such a measure would allow for cross-cultural comparisons in the effects of migraine on quality of life. The aim of this study was to address the need for a reliable and valid version of the MSQ in an additional languages by translating and then evaluating a Chinese version of the measure.

Based on research that has examined quality of life in migraineurs’ lives [7–9], we identified a number of validated measures assessing domains that would be expected to be significantly associated with the MSQv2.1 scale scores, if the MSQv2.1-C were valid. The specific validity criterion we identified included, first, *pain intensity*, which has been shown to be associated negatively with measures of quality of life in individuals with migraine [8, 10, 11]. Second, *migraine-related disability* has been shown to be negatively associated with health-related quality of life in this population [8, 12–14]. Finally, *migraine attack frequency* has been shown to be associated negatively with health-related quality of life both in migraineurs [8, 11] and chronic migraineurs [9]. Therefore, we included measures of each of these domains (i.e., average and worst pain intensity, disability, and attack frequency) as validity criterion in the current study. We hypothesized that if the translated version of the MSQv2.1 were valid, measures of each of these domains measures would be significantly associated with the MSQv2.1 scale scores.

## Methods

### Design, setting & participants

This study adopted the consecutive sampling method in the neurological outpatient departments of one medical center in northern Taiwan. Eligible participants were migraine patients. Exclusion criteria were: (1) being 19 years old or younger; (2) being 66 years old or older; (3) having rarely experienced migraines; and (4) having migraine-related complications, such as familial hemiplegic migraine (ICHD codes 1.2.4), sporadic hemiplegic migraine (ICHD codes 1.2.5), basilar-type migraine (ICHD codes 1.2.6), childhood periodic syndromes that are commonly precursors of migraine (ICHD codes 1.3), retinal migraine (ICHD codes 1.4) and other migraine complications (ICHD codes 1.5). All eligible patients were referred to the research staff by physicians from the neurological outpatient departments of a medical center in northern Taiwan. Research assistants described the purpose of the study, and interested patients were asked to provide written informed consent.

### Instruments

All participants were administered the MSQ Chinese version, 12-Item Short Form Survey (SF-12), MIDAS, and questions asking about demographic information (age, BMI, education and marriage status). We used a 0–10 numerical rating scale (NRS) to assess the participants’ average and worst pain intensity in the past week. The participants also provided migraine-related information (e.g., if they had migraine with or without aura).

#### Migraine-Specific Quality of Life Questionnaire version 2.1 (MSQv2.1)

The MSQv2.1 measures quality of life among migraine patients during the past 4 weeks. It has three scales assessing three quality of life domains: (1) *Role Restrictive* (RR, original named *Role Function-Restrictive* in MSQv1), which includes seven items that assess how patients’ performance of normal activities is limited by migraine; (2) *Role Preventive* (RP, original named *Role Function-Preventive*), which consists of four items that assess how patients’ performance of normal activities is interrupted by migraines; and (3) *Emotion Function* (EF), which consists of three items that assess the impact of migraine on the respondent’s emotions (e.g., frustration or helplessness). The item responses range from one to six (1 = “None of the time;” 2 = “A little bit of time;” 3 = “Some of the time;” 4 = “A good bit of the time;” 5 = “Most of the time;” 6 = “All of the time”). All items are reverse-coded, and standardized to a 0–100 scale. Thus, higher scale scores indicate better migraine-related quality of life. The original English version of the MSQv2.1 has shown good reliability, including good internal consistency with Cronbach’s alpha ranging from 0.86 to 0.96, and stability with test-retest Pearson’s correlations (*r*) ranging from 0.62 to 0.65 and intra-class correlation coefficients (ICC) ranging from 0.57 to 0.63. The MSQv2.1 has also shown good validity, as indicated by weak to moderate associations with physical component summary (PCS) and mental component scale (MCS) of SF-36 (*r* range = 0.20–0.37) [5], as well as weak to moderate negative associations with migraine symptoms (*r* range = − 0.10 - -0.57) [5].

#### 12-Item Short Form Health Survey (SF-12)

In order to assess the convergent validity of the Chinese version of the MSQ, all participants were administered the SF-12 which assesses a number of function domains. We hypothesized that if the MSQ Chinese version were valid, it would be significantly associated with the SF-12 subscales. The SF-12 [15] is a brief version of the SF-36 and its scores have been shown to satisfactorily reflect the original SF-36 scores [16]. The SF-12 assesses eight function domains: physical functioning, role-physical, role-emotional, mental health, bodily pain, general health, vitality, and social functioning. These eight domains can be summarized into two primary summary scores assessing physical functioning (i.e. physical component summary score, PCS) and psychological functioning (i.e., a mental component summary, MCS). The SF-12 has demonstrated strong psychometric properties, including good test-retest reliability and validity in a variety of patient populations [17–19].

#### Migraine Disability Assessment Scale (MIDAS)

Migraine-related disability was assessed using the 5-item Migraine Disability Assessment Scale (MIDAS) [20]. Using a referent period of previous 3 months, the MIDAS items assess respondents’ disability in three areas: work/school, household work, and social activity. The total MIDAS score can range from 0 to 270 with the following disability classification criteria: (1) 0 to 5: slight or no disability, (2) 6 to 10: low levels of disability, (3) 11 to 20: moderate disability, and (4) 21 or above: severe disability. The MIDAS has demonstrated adequate 21-day test-retest reliability (Spearman’s rho) ranging from 0.67 to 0.73 for individual items and good test-retest reliability (Spearman’s rho = 0.84) for overall scores [20]. Moreover, the MIDAS has evidenced good internal consistency (Cronbach’s α value of 0.83) [20]. The MIDAS scores have also been shown to be moderately correlated with pain intensity scores extracted from a pain dairy (*r* = 0.63), supporting its convergent validity [21]. Finally, MIDAS scores have been shown to be significantly different between migraineurs and non-migraineurs, supporting its discriminant validity [20].

### Translation process and psychometric testing

Three phases were implemented in to develop and evaluate the Chinese version of the MSQ. First, the MSQv2.1 was translated into traditional Chinese by a rigorous translation process [22, 23], including forward translation and back translation, to establish semantic and content equivalencies. Second, the MSQv2.1 Chinese version was administrated to 11 monolingual migraine patients to evaluate its readability and understandability. Third, we evaluate the psychometric properties of the measure in a sample of monolingual individuals with migraine to evaluate its reliability and validity.

### Phase I: forward translation and back translation process

We recruited four bilingual experts for the forward and backward translation phase; three have a Ph.D. degree, and one a university lecturer. The bilingual experts worked independently. We also recruited a native speaker, who is an experienced English language editor, to compare the meaning of original and back-translated English versions in order to ensure the harmonization of the translations.

After forward and backward translation of the MSQ instructions and items by the bilingual and native speakers, we evaluated the readability and understandability of translated words via cognitive testing of the instructions and items with 11 monolingual migraine patients. Their comments were used to modify the wording as needed to improve the readability and understandability of the translation.

### Phase II: psychometric testing

We computed Cronbach’s *α* values to evaluate the internal consistency for the entire scale and subscales. Cronbach alpha’s 0.7 or larger were used to indicate acceptable internal consistency [24]. We also computed Spearman’s ρ correlation and intra-class correlation coefficients (ICCs) using a test-retest period of 3 days to evaluate the scales’ reliability. Spearman’s ρ correlation coefficients 0.70 or larger were deemed to indicate acceptable test-retest reliability [25]. ICC cutoffs used to indicate poor, moderate, good, and excellent reliability were < 0.50, 0.50 to 0.75, 0.75 to 0.90, and greater than 0.90, respectively [26].

In order to evaluate construct validity, we conducted a confirmatory factor analysis (CFA) with maximum likelihood estimation (MLE) to examine the factor structure of the items (in terms of indicator loadings) and model fit indices. Indicator loadings (λ) > .50 were used to indicate acceptable levels of construct validity (i.e., items’ variance explained by the construct) [27]. Cut-off points indicating acceptable fit for each fit index were as follows: non-normed fit index (NNFI), > .90; comparative fit index (CFI), > .90, and incremental fit index (IFI), >.90 [28].

We also examined convergent validity by computing Spearman’s ρ correlation coefficients between the MSQv2.1-C and SF-12 scale scores. Furthermore, we evaluated criterion validity (i.e., “the general term to describe how scores on one measure predict on another measure of interest”) [29] by computing Spearman’s ρ correlation coefficients between the MSQv2.1-C scores and the measures of pain intensity, frequency, and migraine-related disability (i.e., MIDAS). We anticipated that, if valid, the MSQv2.1-C score would have statistically significant but weak to moderate associations (i.e., ρs between 0.3 and 0.6) [30] with pain intensity, pain frequency, and disability (MIDAS scores). We use LISREL 8.80 to conduct the CFA, and SPSS 21.0 to conduct all other analyses. Significance level was set at 0.05. Two-tailed tests were used.

### Ethical considerations

Prior to participant enrollment and data collection, we obtained the approval by the institutional review board (IRB) of the National Taiwan University Hospital (approval number 201505065RIND). Written informed consent was obtained from all participants prior to data collected. Consented participants were asked to complete the study questionnaire. Three days later, the participants were invited to complete the MSQv2.1-C again for evaluated the test-retest reliability. Participants were offered NT$100 (about US$3.30) gift certificates as compensation for their participation. The survey study that was the source of the data for the current analyses provided data that were also used to investigate the frequency and perceived efficacy of pain management strategies among the participants. The findings from the analyses using the data assessing the use of pain management strategies will be presented elsewhere.

## Results

### Description of sample

We recruited 174 participants into the current study. Table 1 lists description information for the study sample. As can be seen, the participants had an average age of 38.5 years with a standard deviation (SD) of 11.8 years. The majority of participants was female (87%) and married (53%) and had attended college or university (66%). Regarding clinical characteristics, 70% participants’ migraine type was without aura and had an average headache pain intensity of 5.5 (SD = 2.1), and a worst pain of 6.3 (SD = 2.1) between 0 and 10, numerical rating scale. Their Migraine Disability Assessment Scale (MIDAS) scores indicated that in the past 3 months migraine tended to interfere infrequently with their daily activities (48%). MSQv2.1-C scale scores from the study sample are presented in Table 2.

**Table 1:** Participant Profile in Demographics and Pain-related Items

Variables	All Participants (*n* = 174)
Age, mean (SD), y	38.5 (11.8)
20–30, No. (%)	49 (28)
31–40, No. (%)	57 (33)
41–50, No. (%)	35 (20)
51–60, No. (%)	26 (15)
60–65, No. (%)	6 (3)
Missing (refuse to provide)	1 (1)
Female, No. (%)	152 (87)
Body mass index, mean (SD)	22.6 (3.9)
Range	15.0–40.1
Education, No. (%)	
High school or below	32 (18)
College	115 (66)
Graduate school	27 (16)
Marriage status, No. (%)	
Unmarried	75 (43)
Married	92 (53)
Ever married	7 (4)
Family income, New Taiwan Dollars	
Below 19,999, No. (%)	12 (7)
20,000-59,999, No. (%)	71 (41)
60,000-99,999, No. (%)	48 (28)
Above 100,000, No. (%)	37 (21)
Missing (refuse to provide), No. (%)	6 (3)
Migraine with aura, No. (%)	52 (30)
Average pain intensity, mean (SD)	5.5 (2.1)
Mild (1–4), No. (%)	55 (32)
Moderate (5, 6), No. (%)	59 (34)
Severe (7–10), No. (%)	60 (34)
Worst pain intensity, mean (SD)	6.3 (2.1)
Mild (1–4), No. (%)	33 (20)
Moderate (5, 6), No. (%)	47 (28)
Severe (7–10), No. (%)	85 (52)
Disability (MIDAS), mean (SD)	16.0 (33.3)
Minimal or infrequent (0–5), No. (%)	84 (48)
Mild (6–10), No. (%)	32 (18)
Moderate (11–20), No. (%)	27 (16)
Severe (21 or above), No. (%)	31 (18)

*Abbreviation*: *MIDAS* migraine disability assessment scale

**Table 2:** Summary of confirmatory factor analysis and reliability

Construct-item	μ	SD	λ	*M*	*SD*	Range	α^a^	ICCs	ρ
Role Restrictive				67.7	19.0	11–100	0.93	0.72	0.74
MSQ01	4.50	1.092	0.83						
MSQ02	4.27	1.172	0.86						
MSQ03	4.61	1.055	0.88						
MSQ04	4.69	1.032	0.85						
MSQ05	4.32	1.083	0.85						
MSQ06	4.23	1.216	0.85						
MSQ07	4.08	1.253	0.80						
Role Preventive				80.0	17.1	10–100	0.86	0.75	0.78
MSQ08	5.02	0.931	0.88						
MSQ09	5.06	1.049	0.82						
MSQ10	5.02	0.982	0.91						
MSQ11	4.90	1.049	0.81						
Emotion Function				80.4	20.0	0–100	0.81	0.69	0.76
MSQ12	4.43	1.272	0.71						
MSQ13	5.29	1.136	0.96						
MSQ14	5.35	1.103	0.88						

*Abbreviation*: *α* Cronbach’s *α* value, *λ* indicator loading coefficient, *μ* mean value, *ρ* Spearman’s ρ correlation coefficient

^a^The Cronbach’s *α* for the entire scale (14 items) is 0.95

### Reliability

#### Internal consistency

Table 2 presents the results of the internal consistency analyses. As can be seen, all three of the MSQv2.1-C scale scores had at least good internal consistency, with the Cronbach’s α values being ≥ 0.81.

#### Stability (test-retest reliability)

We collected 113 test-retest responses. The Spearman’s ρ correlations between the test-scores and the retest-scores are presented in Table 2, and are 0.74 for Role Restrictive, 0.78 for Role Preventive, and 0.76 for Emotion Function. The ICC values were 0.72 (for Role Restrictive), 0.75 (for Role Preventive), and 0.69 (for Emotion Function).

### Construct validity

To test construct validity, we conducted a confirmatory factor analysis using LISREL v.8.8. The results are presented in Table 2. All indicator loadings (λ) exceeded 0.50, confirming the adequacy of the factor structure [31]. Moreover, the results indicate an adequate fit for a 3-factor structure; non-normed fit index = 0.94 (> 0.90), comparative fit index = 0.95 (> 0.90), and incremental fit index = 0.95 (> 0.90).

### Convergent validity

The scores of MSQv2.1-C evidence weak to moderate associations the SF-12 scores (the correlation coefficients ranged from 0.27 to 0.37, see Table 3).

**Table 3:** Correlations between MSQ Subscales and Criterions^a^

Criterions	MSQ-Role Restrictive	MSQ-Role Preventive	MSQ-Emotional Function
SF-12			
PCS	.37^**^	.35^**^	.31^**^
MCS	.37^**^	.27^**^	.36^**^
Pain intensity			
Average	−.43^**^	−.37^**^	−.33^**^
Worst	−.40^**^	−.33^**^	−.31^**^
Pain frequency	−.45^*^	−.33^*^	−.31^*^
MIDAS			
Total	−.50^**^	−.44^**^	−.35^**^
Work/School	−.38^**^	−.30^**^	−.27^**^
Household work	−.33^**^	−.34^**^	−.25^**^
Social activities	−.46^**^	−.51^**^	−.41^**^

*Abbreviation*: *MIDAS* migraine disability assessment scale

^*^p < 0.01, ^**^p < 0.001

^a^Numbers in the table are Spearman’s ρ correlation coefficients

### Criterion validity

As hypothesized, scores on the Chinese version of MSQv2.1 had significant but weak to moderate association with pain intensity, pain frequency, and disability (MIDAS scores). As shown in Table 3, scores on the translated measures were also negatively related to the scores on pain intensity (ρs range = − 0.43 to − 0.31, *p* < 0.001), pain frequency (ρs range = − 0.45 to − 0.31, *p* < 0.01), and MIDAS (ρs range = − 0.51 to ~ − 0.25, *p* < 0.001).

## Discussion

This is the first study to translate and evaluate the psychometric properties of a Chinese version of the MSQv2.1. The findings support the reliability and validity of the MSQv2.1-C for assessing the quality of life specific to migraine patients. Our findings contribute to the MSQ literature by providing a useful version for Chinese-speaking populations, enhancing our ability to evaluate migraine-related quality of life in many more individuals with migraine. Moreover, the availability of a Chinese version of the MSQ will allow for the possibility of cross-cultural research studying the quality of life of individuals with migraine headaches.

The MSQv2.1-C demonstrated satisfactory internal consistency (all Cronbach alphas > 0.8) and test-retest reliability (all ICCs ≥0.69, and test-retest stability coefficients ≥0.74), consistent with previous reports that documented reliability of the English version of the questionnaire [5, 32]. Moreover, we used multiple approaches to establish the validity of the MSQv2.1-C, in terms construct validity, convergent validity, and criterion validity. First, by confirmatory factor analysis, all indicator loadings (0.71 to 0.96) were much higher than 0.50 [31], confirming the adequacy of the factor structure. Second, we found that the range of correlations between MSQ and SF-36 (0.27 to 0.37) is consistent with the range of correlations in the literature (i.e., ranging from 0.19 to 0.38; [5], supporting the convergent validity of the MSQv2.1-C. Finally, negative correlations between pain intensity, MIDAS and MSQv2.1 are consistent with the literature (*r* range = − 0.57 to − 0.10) [5], supporting the criterion validity of the Chinese version.

### Limitations and future research

The study has some important limitations that should be considered when interpreting the results. First, we did not evaluate the sensitivity of the MSQv2.1-C to change with treatment. As this is an important component of validity, especially if the measure is to be used to assess outcome in clinical trials, future research is needed to evaluate this important validity criterion. Second, participants were consecutively recruited from an outpatient clinic of a medical center in Taiwan. Therefore, the sample may not reflect the population of people with migraine in Taiwan. Replication of the current findings in additional samples of individuals from Taiwan would be needed to establish the reliability of the findings. Third, all of the measures used in the study are self-report measures, which can increase the strength of the associations found due to method variance. Future researchers evaluating the psychometric properties of the MSQv2.1-C should include objective measures of validity criterion variables, when possible.

Although the sample included individuals with migraine both with aura and without aura, and included individuals with significant variability in disability and pain intensity levels, we did not evaluate the extent to which headache severity or chronicity influenced the MSQv2.1’s psychometric properties. Therefore, the study findings do not specifically address the question of whether the MSQv2.1 is suitable for specific headache subpopulations (e.g., those with severe headache or chronic migraine) of individuals with headaches.

Finally, we measured average and worst pain intensity in the last week to capture participants’ recent pain experiences. However, the MSQv2.1 uses a 4 week timeframe. Thus, it is possible that a subset of participants who have multiple migraine attacks every month may still have not had a headache in the last week. Future studies should adopt a time frame of the last 4 weeks for validity measures when possible when evaluating the MSQv2.1-C.

## Summary and conclusions

Despite the study’s limitations, the findings provide initial support for the reliability and validity of a new version of the MSQ, supporting its potential application in Chinese-speaking migraine populations worldwide. Such applications could facilitate the advancement and dissemination of knowledge regarding quality of life among migraineurs, including research that evaluates similarities and differences in this domain across different cultures.

## Data Availability

Not applicable.

## Abbreviations

EF: Emotion Function
ICC: Intra-class correlation coefficient
ICHD: International Classification of Headache Disorders
MCS: Mental component summary
MIDAS: Migraine Disability Assessment Scale
MSQ: Migraine-Specific Quality of Life
MSQv2.1-C: Migraine-Specific Quality of Life Chinese version 2.1
PCS: Physical component summary score
RP: Role preventive
RR: Role restrictive
SF-12: 12-Item Short Form Survey
